# Ibrutinib in c-MYC and HER2 Amplified Oesophagogastric Carcinoma: Results of the Proof-of-Concept iMYC Study

**DOI:** 10.3390/curroncol29040176

**Published:** 2022-03-22

**Authors:** Fiona Turkes, Annette Bryant, Ruwaida Begum, Michael Davidson, Eleftheria Kalaitzaki, Maria Aresu, Retchel Lazaro-Alcausi, Jane Bryant, Isma Rana, Sue Chua, Lauren Aronson, Sanna Hulkki-Wilson, Charlotte Fribbens, David Watkins, Sheela Rao, Naureen Starling, David Cunningham, Irene Y. Chong, Ian Chau

**Affiliations:** 1 Royal Marsden NHS Foundation Trust, Downs Road, Surrey SM2 5PT, UK; fiona.turkes@rmh.nhs.uk (F.T.); annette.bryant@rmh.nhs.uk (A.B.); ruwaida.begum@rmh.nhs.uk (R.B.); michael.davidson@rmh.nhs.uk (M.D.); eleftheria.kalaitzaki@rmh.nhs.uk (E.K.); maria.aresu@rmh.nhs.uk (M.A.); retchel.lazaro-alcausi@rmh.nhs.uk (R.L.-A.); jane.bryant@rmh.nhs.uk (J.B.); isma.rana@rmh.nhs.uk (I.R.); sue.chua@rmh.nhs.uk (S.C.); sanna.hulkki-wilson@rmh.nhs.uk (S.H.-W.); charlotte.fribbens@rmh.nhs.uk (C.F.); david.watkins@rmh.nhs.uk (D.W.); sheela.rao@rmh.nhs.uk (S.R.); naureen.starling@rmh.nhs.uk (N.S.); david.cunningham@rmh.nhs.uk (D.C.); 2 Institute of Cancer Research, 237 Fulham Road, London SW3 6JB, UK; lauren.aronson@icr.ac.uk (L.A.); irene.chong@icr.ac.uk (I.Y.C.)

**Keywords:** oesophagogastric cancer, HER2, c-MYC, ibrutinib

## Abstract

Oesophagogastric (OG) cancer is a highly lethal disease requiring novel treatment options. c-MYC and/or HER-2 amplified oesophageal cancer models have demonstrated sensitivity to BTK inhibition with ibrutinib. We evaluated the safety and efficacy of ibrutinib in patients with c-MYC and/or HER2 amplified pre-treated advanced OG cancer. c-MYC and HER2 amplification status were determined by FISH. The primary endpoint was overall response rate (ORR). Secondary endpoints were disease control rate (DC) at 8 weeks, safety, progression-free survival (PFS) and overall survival (OS). Eleven patients were enrolled. Eight patients had c-MYC amplified tumours, six were HER2 amplified and three were c-MYC and HER2 co-amplified. Grade ≥ 3 adverse events were fever, neutropenia, and vomiting. Grade ≥ 3 gastrointestinal haemorrhage occurred in three patients and was fatal in two cases. Among seven evaluable patients, three patients (43%) achieved a best response of SD at 8 weeks. No PR or CR was observed. Disease control was achieved for 32 weeks in one patient with a dual c-MYC and HER2 highly co-amplified tumour. The median PFS and OS were 1.5 (95% CI: 0.8–5.1) and 5.1 (95% CI: 0.8–14.5) months, respectively. Ibrutinib had limited clinical efficacy in patients with c-MYC and/or HER2 amplified OG cancer. Unexpected gastrointestinal bleeding was observed in 3 out of 8 treated patients which was considered a new safety finding for ibrutinib in this population.

## 1. Introduction

Gastric and oesophageal (OG) cancers are among the most lethal malignancies worldwide representing the 4th and 6th leading causes of cancer-related death [1]. Chemotherapy and anti-PD1 antibody combinations represent a paradigm shift in the initial management of advanced OG cancer, but beyond the first-line, options remain limited. Median overall survival from second-line chemotherapy is only 5 months [2] and phase III studies evaluating chemotherapy combined with targeted agents have largely yielded negative results [3,4,5,6,7]. The VEGFR2 antibody ramucirumab is approved as a second-line agent alone or in combination with paclitaxel; however, its widespread use is restricted by a lack of funding, for example, in the U.K. Similarly, the antibody-drug conjugate trastuzumab deruxtecan is approved for patients with HER2 positive disease (approximately 20%) beyond the first line in the U.S. and Japan only. Thus far, clinical benefit from second-line anti-PD1 monotherapy is limited to patients with squamous cell pathology [8,9]. There remains significant global unmet need for novel therapies in the treatment of this disease.

Dysregulation of the MYC proto-oncogenes (amplification or chromosomal translocation) that encode for transcription factors frequently present in human cancers [10]. C-MYC plays an important role in controlling carcinogenic processes such as cell growth, differentiation and apoptosis and is amplified in 10–30% of oesophageal carcinomas [11,12]. Dysfunctional genes which code for transcription factors that drive tumour progression are difficult to target directly. In such tumours, it may be appropriate to adopt a synthetic lethal approach, where expression of a combination of two or more aberrant genes leads to cell death, rather than direct targeting with small molecule inhibitors or monoclonal antibodies [13]. Functional genomics screening using isogenic cell line models identified Bruton’s tyrosine kinase (BTK) as having synthetic lethal interactions with c-MYC overexpression. In addition, c-MYC amplified oesophageal cancer cell lines were observed to be synthetically lethal with silencing of BTK [14,15]. Furthermore, BTK-inhibition with ibrutinib, an irreversible selective inhibitor of BTK, has been shown to directly inhibit growth of c-MYC and/or HER-2 amplified oesophageal cancer cell lines with downregulation of c-MYC and p-ERK, as well as corresponding cell-cycle arrest [15]. Ibrutinib is a commonly utilised drug in the treatment of haematological malignancies. These data support the potential of re-purposing BTK inhibition as a novel therapeutic strategy in c-MYC and/or HER2 amplified OG cancer.

This proof-of-concept study assessed the efficacy and safety of ibrutinib monotherapy in previously treated patients with c-MYC and/or HER2 amplified advanced OG cancer.

## 2. Materials and Methods

### 2.1. Patient Eligibility

Adult patients with histologically proven advanced, metastatic, or locally advanced inoperable oesophagogastric squamous or adenocarcinoma with disease progression after at least one prior line of chemotherapy for advanced disease were eligible. In the case of HER2 positive tumours, previous treatment with chemotherapy with or without a HER2 targeted agent were enrolled. Patients were required to have at least one measurable target lesion as per RECIST 1.1. criteria and a World Health Organisation (WHO) performance status of 0–2. Provision of fresh or archival tumour tissue to determine c-MYC or HER2 gene amplification was mandatory. Exclusion criteria included clinically significant cardiovascular disease or known brain metastases. Exclusion criteria were updated during recruitment to exclude any actively bleeding tumour or any prior or current therapeutic anticoagulant treatment.

### 2.2. Study Design

This was an open-label, single-arm phase II study. Pre-screening for c-MYC and HER2 status was performed for patients treated with any line of therapy who the investigator considered might have become eligible on disease progression during the recruitment period (stage 1: pre-screening). A tumour biopsy (either archival or, if not available, fresh), was provided for FISH/CISH testing and a blood sample was also taken to assess c-MYC copy number variation in plasma. Those who were eligible for the study, as per their c-MYC or HER2 status, subsequently provided consent to the main study (stage 2: main study). Patients received 560 mg ibrutinib orally once daily on a 28-day cycle and could continue until disease progression, unacceptable toxicity, or study withdrawal. Patients could receive up to two dose reductions for adverse events (AEs).

### 2.3. Definition of c-MYC or HER2 Gene Amplification

Prior to enrolment in the main study, all patients gave consent to provide archival or fresh tumour tissue for assessment of c-MYC and/or HER2 gene amplification. FISH is a sensitive and specific technique to identify c-MYC and/or HER2 amplification in tumour specimens [16,17]. A dual probe FISH assay was specifically designed and validated to determine tumour MYC amplification in OG cancer using probes from Vysis (Abbott Molecular, Maidenhead, UK). Screening was performed using a combination of a reference probe mapping to the centromere of chromosome 8 (CEP8) and a MYC probe mapping to chromosome 8q24, covering the entire coding region of the MYC gene from exons 1–3. The dual probe approach was used to distinguish between increased copies of chromosome 8 and true MYC amplification. A tumour was considered as having c-MYC amplification on the basis of c-MYC:CEP8 FISH ratio ≥ 2.5 in line with the established HER2 positive threshold by FISH testing in breast cancer [18]. Patients whose tumours demonstrated c-MYC amplification and a c-MYC ratio of ≥2.5 were considered eligible. Patients potentially eligible for main study entry were also considered for HER2 amplification screening. However, central HER2 testing was not mandatory if locally performed HER2 results were available. Tumours were considered HER2 amplified as per ASCO-CAP guidelines. 

### 2.4. Study Assessments and Toxicity

Baseline imaging was performed during screening, at week 8 after starting treatment and every 8 weeks thereafter until disease progression. FDG-PET scans were performed prior to starting ibrutinib, at day 14 and at 8 weeks during treatment. Toxicity was assessed according to the Common Terminology Criteria for Adverse Events version 4.0. Safety data were summarised according to grade and time point.

### 2.5. Upper Gastrointestinal Bleeding Episodes Observed on Study

After recruiting and treating 5 patients within the study, 3 episodes of upper gastrointestinal bleeding were observed, 2 of which proved fatal. Recruitment to the main study was paused from January 2018 until the IDMC could convene to review this emergent safety concern. The study reopened in May 2018 after a substantial amendment was approved which excluded patients with prior or current anticoagulation treatment and recommended that ibrutinib should be discontinued for a minimum of 7 days if an oesophageal stenting procedure was required while on study. Patients with their primary tumour in situ who had previously undergone an oesophageal stenting procedure were also excluded going forward.

### 2.6. Outcomes

The primary endpoint was overall response rate (ORR) defined as CR and PR (assessed according to RECIST 1.1) at 8 weeks. Secondary endpoints were disease control rate (DC), defined as CR, PR, or SD at 8 weeks and PFS (defined as time from the start of study treatment to progression or death from any cause) and OS.

### 2.7. Statistical Plan

The planned sample size was between 9–17 evaluable patients. To assess the primary endpoint assuming that ibrutinib is active with a true underlying response rate of >20%, the chance of seeing no responses in a cohort of 17 patients would be low (approximately 3%, calculated on binomial probabilities). A Simon two-stage design was planned to be incorporated for primary endpoint analysis as follows: if 1 or more patients in the first 9 demonstrate a response, then the study may expand to 17 patients. If no patient in the first 9 demonstrates a response, then study closure would be considered. If 3 or more patients out of 17 demonstrate a response, then further research would be indicated. This gives 80% power with an alpha of 5% to detect a 25% response rate while ruling out a 5% activity rate. Within the first projected recruitment of 9 patients, at least 4 were to demonstrate c-MYC amplification. The remaining 5 would show either c-MYC or HER2 amplification or co-amplification of both. 

However, due to slow recruitment, the COVID-19 pandemic and no further pre-screening capacity, the Trial Management Group made the decision to close the trial in June 2020. Patients who were pre-screened during their previous line of treatment and potentially eligible at that time could no longer be considered for the main study as they were either deceased, receiving other treatment, or had commenced anticoagulation due to thrombosis which became an exclusion criterion. The final analysis was thus performed after 8 patients had received treatment. 

## 3. Results

### 3.1. Patient Characteristics

**Figure 1:**
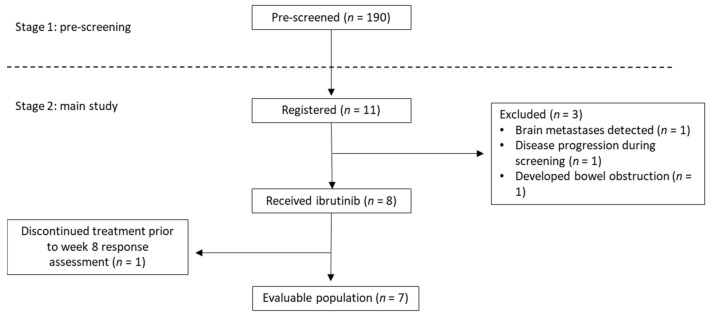
Consort diagram.

Between July 2016 and December 2019 190 patients were enrolled into stage 1: pre-screening. Of 190 pre-screened patients, 11 patients were registered on the main study and 8 patients received treatment with ibrutinib (Figure 1). One patient died before having the first response assessment and therefore, was not evaluable for the primary endpoint. Patients’ characteristics are provided in Table 1. The median age was 63 years (range 58–69 years), and most patients were male (82%). Most patients’ tumours were adenocarcinoma (73%) rather than squamous carcinoma (27%) and half were HER2 positive (55%). Most registered patients were pre-screened during their first-line treatment (45%) or shortly after completion of their first line (27%). Most patients had liver metastases (80%), three patients had lymph node metastases, two patients had lung metastases and one patient had bone metastases. Eight registered patients had tumours which were c-MYC amplified, six were HER2 amplified and three were c-MYC and HER2 co-amplified. Five out of eight patients who started ibrutinib were HER2 positive (two of these were c-MYC and HER2 co-amplified). All except two patients had received trastuzumab in the first-line setting. The two patients who were not previously treated with trastuzumab experienced disease progression during or within 6 months of neoadjuvant/adjuvant therapy or definitive chemoradiation, and therefore, their neoadjuvant/adjuvant therapy was counted as first-line treatment of advanced disease.

Of the eight patients who received study treatment, no dose reductions were required. However, two patients (67%) had a dose interruption at cycle four.

### 3.2. Therapeutic Response

**Figure 2:**
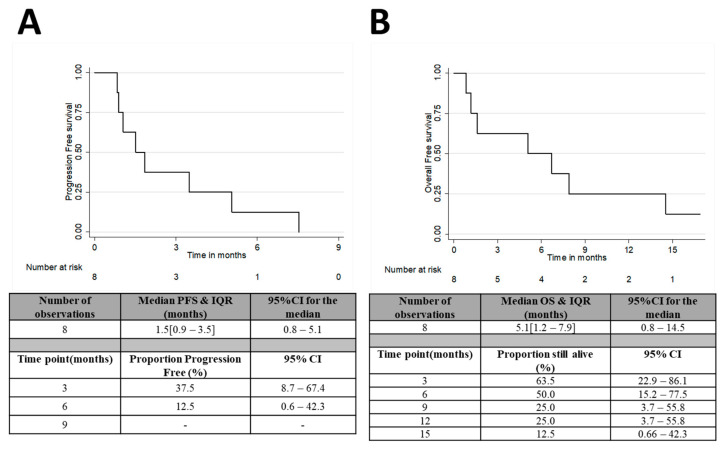
(**A**) Progression free survival and (**B**) overall survival in the treated population.

After 8 weeks of treatment, the best ORR rate (CR/PR) was 0% in the evaluable population and treatment failed in 4 patients (57%). The DC rate after 8, 16 and 24 weeks was 43%, 29% and 14%, respectively (Table 2). Of the three patients (43%) with SD after two cycles, one patient whose tumour was co-amplified for c-MYC and HER2 went on to achieve disease control for 32 weeks after nine cycles of treatment with a partial metabolic response in the primary tumour on serial PET–CT scans (Figure S1). All patients were off treatment at the time of analysis and all but one patient was deceased. The median number of cycles received was 2 (IQR 1–4). Six patients (75%) stopped ibrutinib due to disease progression, one patient stopped due to toxicity and one patient died while on treatment. Median PFS was 1.5 months (95% CI; 0.8–5.1) and the median OS was 5.1 months (95% CI; 0.8–14.5) (Figure 2A,B).

### 3.3. Treatment Tolerance and Toxicity

The most common adverse events were anaemia, constipation, diarrhoea, fever, vomiting and fatigue in line with toxicities known to be associated with ibrutinib (Table 3). Grade ≥ 3 AES were fever, neutropenia, and vomiting. Eleven serious adverse events were reported in patients who received ibrutinib during the study including gastrointestinal haemorrhage (*n* = 3) which resulted in death in two cases. All three of these patients had their primary tumours in situ and two were on therapeutic anticoagulation prior to starting ibrutinib. Of these, the patient who died had a pre-existing oesophageal stent in situ. The third patient had an oesophageal stent inserted after an episode of haematemesis during cycle five of ibrutinib. The patient died shortly after receiving treatment for a chest infection with evidence of recent upper gastrointestinal bleeding. The last dose of ibrutinib had been 5 weeks previously.

## 4. Discussion

To our knowledge, this is the first clinical trial to assess the role of ibrutinib monotherapy in patients with c-MYC and/or HER2 amplified OG cancer. Due to the COVID-19 pandemic and loss of pre-screening capacity in our cytogenetics department to undertake FISH testing, the trial was closed early. Despite compelling pre-clinical rationale harnessing bench-to-bedside research and an opportunity to re-purpose an existing approved drug, ibrutinib did not demonstrate radiological tumour shrinkage as per RECIST 1.1 in pre-treated patients with c-MYC and/or HER2 amplified advanced OG cancer. Although no CR or PR was achieved, three patients (43%) experienced disease stabilisation after 8 weeks. Given that standard second-line chemotherapy improves survival by approximately 6 weeks [2] and only 12–20% of tumours reach a best response of PR after treatment with established second- or third-line regimens [19], it may have been ambitious to aim for response rates (CR/PR) of >20% in our small sample size. We did not collect enough health questionnaires to meaningfully confer quality of life benefit from ibrutinib as only four patients completed them at the start of treatment and most patients progressed after only two cycles. However, it is noteworthy that baseline tumour assessment scans took place up to 28 days before patients started ibrutinib as this is a relatively long time in such a poor prognosis disease. Therefore, the progressions seen at the first-response assessment may have represented the fast pace of the disease process rather than ineffective treatment. In future therapeutic development, achieving disease stabilisation in this patient population might be a clinically meaningful goal. 

One patient with a dual c-MYC and HER2 highly co-amplified tumour continued treatment for 32 weeks which may hint at enhanced efficacy of ibrutinib in a co-amplified tumour; however, most patients progressed after 2 cycles (57%). These early progressions may have been because of primary resistance to BTK inhibition, widely described with ibrutinib use in haematological malignancies (around 30%) [20], due to simultaneous activation of other pathways, such as PI3K-AKT signalling [21]. This raises the possibility of the need for combination therapy targeting more than one intersecting pathway. Furthermore, c-MYC amplification appears to be more frequent in distant metastatic disease compared to the primary tumour and thus likely represents an acquired event through tumour evolution [22]. Therefore, targeting a single genetic vulnerability such as c-MYC amplification may not have been sufficient to overcome intrinsic resistance mechanisms. Pre-clinically, the greatest sensitivity to ibrutinib was also seen in the co-amplified oesophageal models [15] and thus future research may focus on evaluating ibrutinib efficacy in dual c-MYC and HER2 highly co-amplified tumours (3/7, 43% of the evaluable population in this study). 

Our group observed considerable inter- and intra-tumoral heterogeneity between patterns of MYC amplification in the pre-screened study population [23] in line with previous findings in MYC amplified gastric cancer [12]. This may have contributed to the varied responses seen in this study. Additionally, the cut-off for c-MYC ‘positivity’, selected based on parameters for HER2 FISH testing in breast cancer, may not have been set sufficiently high enough to overcome this therapeutic hurdle. Indeed, breast cancers with any degree of HER2 amplification appear to demonstrate sensitivity to HER2 targeted therapy; [24] however, much higher levels of HER2 or FGFR2 expression are required before effective therapeutic targeting is seen in gastric cancer [25,26]. Furthermore, while the c-MYC and/or HER2 oesophageal cancer cell lines were sensitive to ibrutinib at nanomolar concentrations [15], cell lines do not account for histological obstacles posed by the complex architecture of human cancers. Future work might seek to evaluate the magnitude of this synthetic lethal effect in humanised mouse models. The timing of the tissue sampling used to determine c-MYC/HER2 status may have also contributed to the relative lack of efficacy seen. For example, HER2 positivity has been shown to disappear in two thirds of patients on repeat biopsy after first-line therapy compared to before treatment [27]. As pre-screening was mostly performed on archival diagnostic biopsy samples (7/8 treated patients (88%)), this may not have been reflective of the biology of the patients’ disease at the time of study enrolment. A limitation of our study was that relatively few sequential tumour biopsies were collected during the study, precluding insight into dynamic changes of amplification status at baseline and progression which may have corresponded with treatment response and resistance. The heterogeneity and dynamic status of c-MYC amplification and representative and reproducible biomarker determination will be crucial issues to address in future attempts to target c-MYC.

The adverse event profile in this study was consistent with previous studies of ibrutinib apart from the 3 out of 8 patients who suffered gastrointestinal bleeding. Since these episodes were observed on the iMYC trial, safety warnings for ibrutinib now include major bleeding events such as gastrointestinal haemorrhage (risk of any grade haemorrhage with ibrutinib is 32% and ≥grade 3 is 1%) [28]. A phase III study of ibrutinib combined with chemotherapy in patients with advanced pancreatic cancer has also since reported major haemorrhage in 6% of patients [29]. Given the high incidence of gastrointestinal bleeding and associated mortality in this patient population [30], it is important to be mindful of the bleeding risks of such drugs in future study design.

## 5. Conclusions

Single agent ibrutinib had limited clinical efficacy in patients with c-MYC and/or HER2 amplified OG cancer in this proof-of-concept study. However, c-MYC and/or HER2 are important genetic cancer drivers, and the success of future therapeutic efforts will likely require novel drug development as well as the assessment of drug combinations.

## Figures and Tables

**Table 1:** Characteristics of 190 patients who were pre-screened for a *c-MYC* and/or *HER2* amplification.

	Pre-Screened (*n* = 190)	Registered (*n* = 11)	Patients Starting Ibrutinib (*n* = 8)
Age at registration, (years)			
Median (IQR)	NA	63 (58–69)	62 (58–69)
Mean (SD)	NA	62.3 (9.8)	63.5 (8.4)
Min–Max	NA	43–79	52–79
	**N (%)**	**N (%)**	**N (%)**
Gender			
Female	40 (21)	2 (18)	1 (13)
Male	150 (79)	9 (82)	7 (87)
Histology			
Adenocarcinoma	155 (82)	8 (73)	5 (62)
Squamous carcinoma	33 (17)	3 (27)	3 (38)
Mixed	2 (1)	0 (0)	0 (0)
HER2 status			
Negative	154 (81)	5 (45)	3 (38)
Positive	36 (19)	6 (55)	5 (62)
Cmyc status			
Amplified	43 (23)	8 (73)	6 (75)
Not amplified	120 (63)	2 (18)	1 (12)
Failed Testing	25 (13)	1 (9)	1 (12)
Other		-	-
No tumour	1 (1)	-	-
Not tested	1 (1)	-	-
Coamplified	8 (2)	3 (27)	3 (38)
Tumour location			
GOJ	60 (31)	4 (36)	2 (25)
Gastric	40 (21)	1 (9)	1 (13)
Oesophagus	90 (48)	6 (55)	5 (63)
Disease status at time of screening			
First-line on treatment	85 (45)	5 (45)	4 (50)
First-line completed treatment	40 (21)	3 (27)	2 (25)
Second-line on treatment	27 (14)	1 (9)	1 (12)
Second-line completed treatment	21 (11)	1 (9)	1 (12)
Third-line on treatment	8 (4)	0 (0)	0 (0)
Third-line completed treatment	9 (5)	1 (9)	0 (0)

**Table 2:** Best overall response rate in the evaluable population *n* = 7 at 8 weeks and ORR at 16, 24 and 32 weeks.

8 Weeks (Best Response)			16 Weeks		24 Weeks		32 Weeks	
RECIST Response	**N**	**%**	**N**	**%**	**N**	**%**	**N**	**%**
CR	0	0	0	0	0	0	0	0
PR	0	0	0	0	0	0	0	0
SD	3	43%	2	29%	1	14%	0	0
PD	4	57%	5	71%	6	86%	7	100%

**Table 3:** Frequency of maximum grade of each toxicity in the treated population (*n* = 8).

Toxicity	Grade				
	1	2	3	4	5
	**N (%)**	**N (%)**	**N (%)**	**N (%)**	**N (%)**
Anaemia	1 (13)	6 (75)			
Arrhythmia	1 (13)				
Constipation	2 (25)	1 (13)			
Diarrhoea	3 (38)				
Dry skin	2 (25)				
Fatigue	5 (63)	1 (13)			
Fever	2 (25)		1 (13)		
Infection	1 (13)				
Mucositis	1 (13)				
Nausea	1 (13)	1 (13)			
Neutropenia			1 (13)		
Pruritis	1 (13)				
Skin rash/desquamation	1 (13)				
Thrombocytopenia	1 (13)				
Vomiting	1 (13)	1 (13)	1 (13)		

## Data Availability

Access to the anonymised dataset can be requested from the corresponding author.

## Supplementary Materials

The following supporting information can be downloaded at: https://www.mdpi.com/article/10.3390/curroncol29040176/s1, Figure S1: Sequential PET-CT scan images from a study patient showing reducing FDG-avidity (SUVmax) in the primary oesophagogastric tumour during ibrutinib treatment.
