# Supplementary material for: Ibrutinib in c-MYC and HER2 Amplified Oesophagogastric Carcinoma: Results of the Proof-of-Concept iMYC Study
Source: Curr Oncol. 2022 Mar 22;29(4):2174–84. doi: 10.3390/curroncol29040176 (PMC9029374; doi:10.3390/curroncol29040176)
Supplement: Supplementary file 1 [file curroncol-29-00176-s001.zip › curroncol-1589658-supplementary.pdf]

Current Oncology

Supplementary Material

Ibrutinib in c-MYC and HER2 Amplified Oesophagogastric Carcinoma: Results of the Proof-of-Concept iMYC Study

Fiona Turkes [1], Annette Bryant [1], Ruwaida Begum [1], Michael Davidson [1], Eleftheria Kalaitzaki [1], Maria Aresu [1], Retchel Lazaro-Alcausi [1], Jane Bryant [1], Isma Rana [1], Sue Chua [1], Lauren Aronson [2], Sanna Hulkki-Wilson [1], Charlotte Fribbens [1], David Watkins [1], Sheela Rao [1], Naureen Starling [1], David Cunningham [1], Irene Y. Chong [2] and Ian Chau [1]

[1] Royal Marsden NHS Foundation Trust, Downs Road, Surrey SM2 5PT, UK; fiona.turkes@rmh.nhs.uk (F.T.); annette.bryant@rmh.nhs.uk (A.B.); ruwaida.begum@rmh.nhs.uk (R.B.); michael.davidson@rmh.nhs.uk (M.D.); eleftheria.kalaitzaki@rmh.nhs.uk (E.K.); maria.aresu@rmh.nhs.uk (M.A.); retchel.lazaro-alcausi@rmh.nhs.uk (R.L.-A.); jane.bryant@rmh.nhs.uk (J.B.); isma.rana@rmh.nhs.uk (I.R.); sue.chua@rmh.nhs.uk (S.C.); sanna.hulkki-wilson@rmh.nhs.uk (S.H.-W.); charlotte.fribbens@rmh.nhs.uk (C.F.); david.watkins@rmh.nhs.uk (D.W.); sheela.rao@rmh.nhs.uk (S.R.); naureen.starling@rmh.nhs.uk (N.S.); david.cunningham@rmh.nhs.uk (D.C.)

[2] Institute of Cancer Research, 237 Fulham Road, London SW3 6JB, UK; lauren.aronson@icr.ac.uk (L.A.); irene.chong@icr.ac.uk (I.Y.C.)

* Correspondence: ian.chau@rmh.nhs.uk

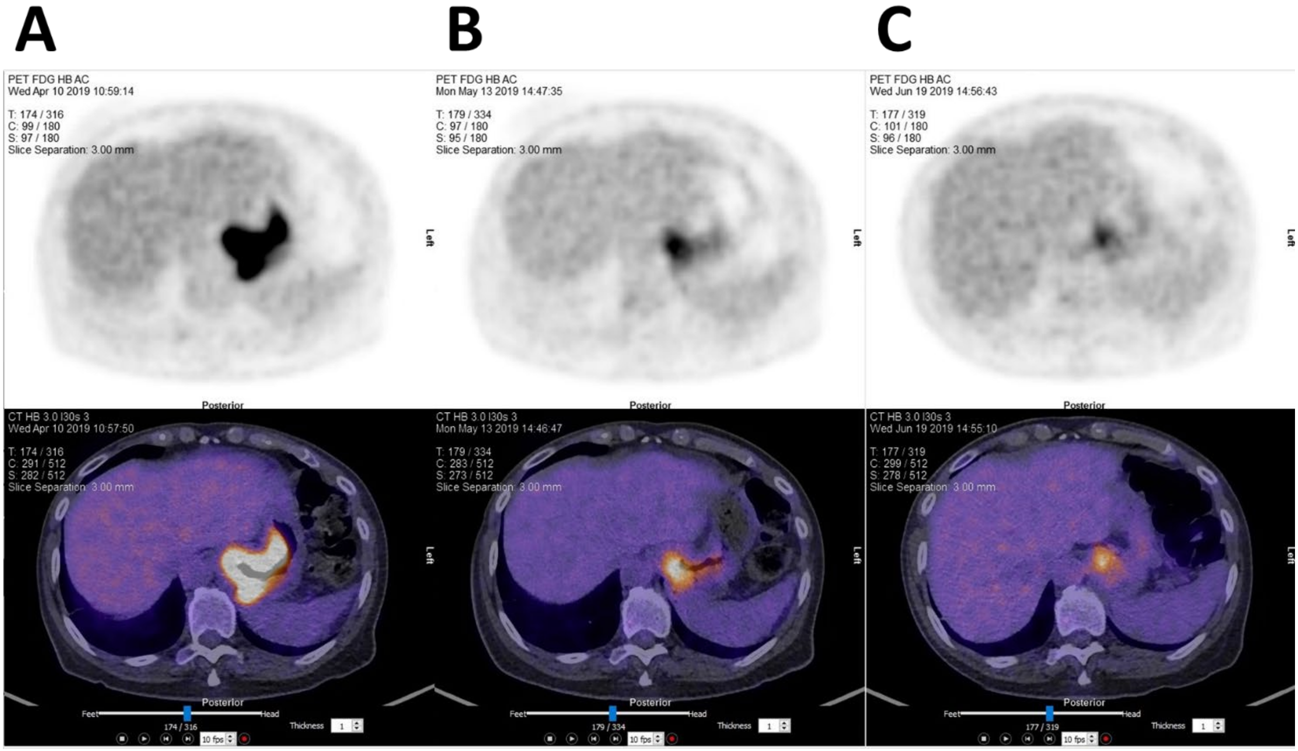

Figure S1. Sequential PET-CT scan images from a study patient showing reducing FDG-avidity (SUVmax) in the primary oesophagogastric tumour during ibrutinib treatment. (**A**) shows the primary tumour in the oesophagogastric junction/cardia at baseline (SUVmax was 14.77), (**B**) at day 14 (SUVmax 7.88) and (**C**) at week 8 (SUVmax 6.47). SUVmax reduced by 56% between baseline and week 8 consistent with a partial metabolic response.
